# Rotavirus vaccine product switch in Ghana: An assessment of service delivery costs, switching costs, and cost-effectiveness

**DOI:** 10.1371/journal.pgph.0001328

**Published:** 2023-08-09

**Authors:** Richmond Owusu, Mercy Mvundura, Justice Nonvignon, George Armah, John Bawa, Kwadwo Odei Antwi-Agyei, Kwame Amponsa-Achiano, Frederick Dadzie, George Bonsu, Andrew Clark, Clint Pecenka, Frederic Debellut

**Affiliations:** 1 School of Public Health, University of Ghana, Accra, Ghana; 2 Center for Vaccine Innovation and Access, PATH, Seattle, Washington, United States of America; 3 Africa Centre for Disease Control and Prevention, Addis Ababa, Ethiopia; 4 Noguchi Memorial Institute for Medical Research, University of Ghana, Accra, Ghana; 5 Center for Vaccine Innovation and Access, PATH, Accra, Ghana; 6 Expanded Programme on Immunization, Ghana Health Service, Accra, Ghana; 7 Department of Health Services Research and Policy, London School of Hygiene & Tropical Medicine, London, United Kingdom; 8 Center for Vaccine Innovation and Access, PATH, Geneva, Switzerland; Bangladesh Institute of Development Studies, BANGLADESH

## Abstract

Ghana introduced rotavirus vaccine (ROTARIX 1-dose presentation) into the routine national immunization program in 2012 and switched to a different product (ROTAVAC 5-dose presentation) in 2020. ROTAVAC has a lower price per dose (US$0.85 versus US$2.15 for ROTARIX) and smaller cold chain footprint but requires more doses per regimen (three versus two). This study estimates the supply chain and service delivery costs associated with each product, the costs involved in switching products, and compares the cost-effectiveness of both products over the next ten years. We estimated the supply chain and service delivery costs associated with ROTARIX and ROTAVAC (evaluating both the 5-dose and 10-dose presentations) using primary data collected from health facilities in six of the 14 regions in the country. We estimated the costs of switching from ROTARIX to ROTAVAC using information collected from key informant interviews and financial records provided by the government. All costs were reported in 2020 US$. We used the UNIVAC decision-support model to evaluate the cost-effectiveness (US$ per disability-adjusted life-year (DALY) averted from government and societal perspectives) of ROTARIX and ROTAVAC (5-dose or 10-dose presentations) compared to no vaccination, and to each other, over a ten-year period (2020 to 2029). We ran probabilistic sensitivity analyses and other threshold analyses. The supply chain and service delivery economic cost per dose was $2.40 for ROTARIX, $1.81 for ROTAVAC 5-dose, and $1.76 for ROTAVAC 10-dose. The financial and economic cost of switching from ROTARIX to ROTAVAC 5-dose was $453,070 and $883,626, respectively. Compared to no vaccination, the cost per DALY averted was $360 for ROTARIX, $298 for ROTAVAC 5-dose, and $273 for ROTAVAC 10-dose. ROTAVAC 10-dose was the most cost-effective option and would be cost-effective at willingness-to-pay thresholds exceeding 0.12 times the national GDP per capita ($2,206 in the year 2020). The switch from ROTARIX to ROTAVAC 5-dose in 2020 was cost-saving. Rotavirus vaccination is highly cost-effective in Ghana. A switch from ROTAVAC 5-dose to ROTAVAC 10-dose would be cost-saving and should be considered.

## Background

Rotavirus remains a public health concern globally, with children in low- and middle-income countries (LMICs), and specifically in sub-Saharan Africa, suffering the brunt of rotavirus morbidity and mortality [1, 2]. Rotavirus was responsible for an estimated 151,514 deaths in children younger than five years old globally in 2019 [3]. In Ghana, diarrhea is the second most common health problem treated in outpatient departments for the population younger than five years old [4]. Before introduction of rotavirus vaccine in 2012, rotavirus accounted for about one in every four diarrhea-related hospitalizations and deaths in this age group [5–7].

Worldwide, vaccination is considered the most effective and cost-effective intervention for reducing rotavirus morbidity and mortality [2, 8–11]. As of September 2022, 115 countries had introduced rotavirus vaccination [12]. This has led to substantial numbers of averted rotavirus hospitalizations and deaths in children younger than five years old [13].

Ghana introduced ROTARIX in 2012 with the support of Gavi, the Vaccine Alliance (Gavi) and has reported high coverage of the last dose, above 90% since 2014 [14]. A prior study showed that rotavirus vaccination (with ROTARIX) was cost-effective in Ghana [11]. Recently, several more rotavirus vaccine products have become available. ROTAVAC received World Health Organization (WHO) prequalification in January 2018 and is currently being used in immunization programs in India, Palestine, Benin, Timor-Leste, and Nigeria [12]. As such, in 2019, Ghana sent a formal request to Gavi to switch from ROTARIX to ROTAVAC. Drivers for this switch included the more affordable price per dose ($0.85 for ROTAVAC versus $2.15 for ROTARIX) and the smaller cold chain footprint per dose (4.2 cm^3^ for ROTAVAC versus 17.12 cm^3^ for ROTARIX) [15].

This switch has implications for Ghana in terms of financing for rotavirus vaccination. Ghana’s co-financing increases every year as Gavi support decreases and Ghana accelerates its transition to fully self-financing vaccines. Further, the switch has implications because of the differences between the two vaccines. For instance, the two rotavirus vaccines differ in several ways, including the number of doses required per child (two doses for ROTARIX versus three doses for ROTAVAC), their presentation in terms of dose per vial (single dose for ROTARIX versus 5- or 10-dose vial for ROTAVAC), and the volume of each presentation. Further, vaccine administration and handling of opened multi-dose vials, which impacts vaccine wastage during service delivery, also has the potential to influence cost and, as a result, cost-effectiveness and immunization financing needs. While single-dose vaccine presentations may minimize vaccine wastage, the cost associated with their procurement and cold chain storage may be higher than with multi-dose vial presentations [16, 17].

We estimated the financial and economic cost of switching products, the economic costs of supply chain and service delivery, as well as the cost-effectiveness of rotavirus vaccination for both vaccines, for 10 years starting from the switch, as part of efforts to update previous cost-effectiveness analyses (CEA) on rotavirus vaccination in Ghana [11, 18].

## Materials and methods

### Estimating the incremental supply chain and service delivery costs for ROTARIX and ROTAVAC in 2020

Incremental cost focuses on additional costs related to rotavirus vaccine delivery, not the entire immunization program. In this study, we collected data to estimate the costs of supply chain and service delivery for the immunization program for 2020, as well as quantities of vaccines used in 2020 and 2021. We did not include any cost resulting from provision of vaccines outside of the scheduled routine vaccination visits. We collected primary data from six out of 14 randomly selected regions of the country (Ashanti, Bono East, Volta, Central, North-East, and Savannah) that cover the three ecological zones, with subsequent randomly sampled districts and health facilities in those regions. Study sites included the Ghana Health Service/Expanded Program on Immunization (GHS/EPI) offices responsible for the overall immunization program management at central level, GHS/EPI offices at the regional and district levels, and health facilities where immunization services are provided. Sampling of these districts and health facilities was done using the Sample Design Optimizer (SDO) tool [19]. A total of 36 health facilities were sampled across 12 districts in the six selected regions. The data collection was done by trained research assistants from University of Ghana through face-to-face interviews with immunization program staff at the study facilities. These interviews were conducted between March and April 2021 and were done using structured costing questionnaires programmed using the electronic data capture software REDCap. REDCap is a free, secure, web-based application designed to support data capture for research studies.

To estimate the incremental economic costs for supply chain and service delivery, the study collected information on the quantities of resources used by the routine immunization program including human resources time use, and resources used for vaccine transportation, vaccine storage, and waste management. We accounted for financial and economic costs of the resources used. Estimation of vaccine storage costs accounted for the annualized capital costs of existing cold chain equipment used for vaccine storage and annual operating cost of the equipment (i.e., energy cost). Capital costs of vehicles and equipment were discounted using a 3% discount rate over their assumed useful life of 10 years. We estimated the cost per cm^3^ of vaccine storage by dividing estimated annual costs for cold chain at each facility by the total vaccine storage capacity of the equipment. Costs were than allocated to the rotavirus vaccines based on their relative cold chain volumes multiplied by the estimated cost per cm^3^.

To estimate vaccine transportation cost, the study team collected information such as the types of vehicles used for vaccine delivery, the expenditures on fuel and maintenance, the number of trips made and distance travelled in a year. We also estimated the annual operating cost for the vehicles (i.e., cost of fuel and maintenance). We estimated the cost per cm^3^ transported by dividing the annual transport costs by the total capacity of vaccines that could be transported per year by each vehicle owned by the facility, using the same assumed frequency of trips observed in the data. When vaccines were not collected together with immunization supplies, the full transport costs of the trip were included. However, when vaccines were reported as collected together with immunization supplies, we allocated the shared transport costs to vaccines based on the volume of the space taken up in the vehicles by vaccines. Immunization supplies are bulkier, with a typical syringe having a volume of 30 cm^3^ compared to vaccines whose volumes typically range from 1 to 4 cm^3^ per dose in multi-dose vials [20] and hence this information was used to determine the allocation factor for transport costs for vaccines.

For estimation of total human resource costs at each level, we multiplied the salary for each staff by the percentage of time spent on immunization program-related activities and aggregated this across the staff at the facility. We obtained data on time spent on immunization program-related activities through the primary data we collected and obtained average salaries by staff level/grade from the Ministry of Health using the government’s salary scale. To estimate cost per minute of staff time at each facility, we divided the total human costs of all staff working in the immunization program at the facility by the estimated total time in minutes spent on immunization related activities.

### Estimating the cost of switching from ROTARIX to ROTAVAC in 2020

We assessed the costs of switching from ROTARIX to ROTAVAC in 2020 by conducting key informant interviews with immunization staff at the GHS/EPI central, regional, and district levels. Interviews were meant to identify the activities that occurred with ROTAVAC introduction and the resources used. We estimated financial and economic costs for each activity or investment. The main cost categories assessed included training of GHS staff, planning and coordination, stakeholders’ engagement workshops, social mobilization, and information, education, and communication (IEC) activities. We retrieved financial costs (per diem, meeting costs, fuel, printing, etc.) from financial reports and invoices, while economic costs were estimated by valuing the staff time spent on the different activities. To estimate the total cost of the switch, data collected in the regions and districts were used to define a cost per surviving infant for stakeholder engagement workshops, social mobilization, and IEC activities; and a cost per community health nurse (vaccinator) for planning committees and trainings. These costs were then applied to each district/region that were not part of the study sample, based on their respective number of surviving infants and community health nurses. Districts and regions where primary data was collected remained with their actual cost collected.

### Cost-effectiveness of ROTARIX and ROTAVAC over the period 2020 to 2029

This analysis utilizes the UNIVAC model version 1.4.29 (https://www.paho.org/en/provac-toolkit). UNIVAC is a Microsoft Excel-based static cohort model developed through a collaboration between the Pan American Health Organization and the London School of Hygiene and Tropical Medicine. UNIVAC is used to generate cost-effectiveness estimates based on defined parameters including disease burden, population distribution, health system costs, vaccine efficacy, coverage, and vaccine program costs. UNIVAC is useful for evidence generation to inform decision makers regarding use of new vaccines, and it has been used in several cost-effectiveness studies to date including for pneumococcal, rotavirus, and human papillomavirus vaccines [21–27].

This study projected the costs and health impact of rotavirus vaccination over a 10-year period starting from 2020 comparing the use of ROTARIX, ROTAVAC 5-dose vial, and ROTAVAC 10-dose vial to a no vaccination scenario. Additionally, we compared both ROTAVAC presentations to use of ROTARIX to model cost-effectiveness of switching vaccines. Ghana currently uses the ROTAVAC 5-dose presentation, however, we also assessed the costs and cost-effectiveness of the ROTAVAC 10-dose presentation in this analysis, as the country also expressed interest in using this product in the future.

We used disability-adjusted life-year (DALY) averted as a measure of health impact of vaccination and calculated the incremental cost-effectiveness ratio as the cost per DALY averted for each vaccine. The analysis is conducted from both the healthcare system (i.e., government) and societal perspectives. The societal perspective accounts for the additional cost that households incurred while seeking care (e.g., out-of-pocket payments for treatment, transportation, productivity losses for caregivers). Health impact of rotavirus vaccination with any rotavirus vaccine were measured using health outcomes such as the number of rotavirus cases, outpatient visits, hospitalizations, deaths, and DALYs averted by vaccination.

#### Disease burden data

Incidence rates for each diarrhea disease event were applied to the younger than five years old population to reflect the burden of rotavirus disease in Ghana pre-vaccination. The incidence of rotavirus gastroenteritis (RVGE) is not available in Ghana so we assumed 10,000 per 100,000 cases in children below five years based on the pooled estimate from a global meta-analysis [28]. The severe case rate in 2003 was 7.1% in Ghana according to the Navrongo Rotavirus Research group [29]. In effect, non-severe RVGE cases constitute 92.9% of all RVGE cases. Using the most recent modelled data for 135 LMICs, annual rotavirus deaths in Ghana without vaccination were estimated at 43 per 100,000 children under five years [1]. Disability weights for DALY calculations come from the 2013 Global Burden of Disease Study [30]. Key inputs that were used to estimate the disease burden are presented in Table 1.

**Table 1:** Study input parameters.

Parameter	Value	Low	High	Source
Annual rate per 100,000 aged <5 years				
Overall RVGE incidence				
RVGE non-severe cases	9,290	6,465	12,930	[28]
RVGE non-severe visits	4,181	2,909	5,819	[11]
RVGE severe cases	710	535	1070	[29]
RVGE severe visits	320	241	481	[31]
RVGE severe hospitalizations	568	428	856	Expert opinion
RVGE severe deaths	43	33	56	[1]
Percentage of rotavirus disease by age				
<1 month	0%			[38]
<2 months	2%			
<3 months	6%			
<6 months	27%			
<1 year	68%			
<2 years	93%			
<3 years	98%			
<4 years	99%			
<5 years	100%			
DALY calculation				
Non-severe RVGE				
DALY weight	0.188	0.125	0.264	[30]
Duration of illness (days)	3	3	7	[39]
Severe RVGE				
DALY weight	0.247	0.164	0.348	[30]
Duration of illness (days)	5	3	7	[39]
Vaccine efficacy, coverage, and timeliness				
Dose 1 efficacy after 1 year of follow-up	50.0%	38.2%	65.3%	[32]
Dose 2 efficacy after 1 year of follow-up	79.0%	75.0%	82.0%	[32]
Dose 3 efficacy after 1 year of follow-up	79.0%	75.0%	82.0%	[32]
Vaccine coverage				
Dose 1	97.0%	82.5%	100.0%	[40]
Dose 2	97.0%	82.5%	100.0%	
Dose 3	97.0%	77.6%	100.0%	
Coverage timeliness				[1, 31]
Dose 1				
Coverage at 1 month	3%			
Coverage at 3 months	93%			
Coverage at 6 months	97%			
Coverage at 12 months	97%			
Dose 2				
Coverage at 1 month	0%			
Coverage at 3 months	54%			
Coverage at 6 months	97%			
Coverage at 12 months	97%			
Dose 3				
Coverage at 1 month	0%			
Coverage at 3 months	9%			
Coverage at 6 months	92%			
Coverage at 12 months	97%			
Vaccine price per course (10-year average)				[15, 41, 42]
ROTARIX	$3.54	$3.28	$5.00	
ROTAVAC 5-dose	$1.59	$1.47	$2.34	
ROTAVAC 10-dose	$1.17	$1.11	$1.80	
International handling (% of vaccine price)	3.50%	2.00%	5.00%	[43]
International delivery (% of vaccine price)	6.00%	2.00%	15.00%	[44]
Vaccine wastage rate				
ROTARIX	5%	2%	8%	
ROTAVAC 5-dose	15%	5%	20%	[17]
ROTAVAC 10-dose	20%	10%	30%	
Cost per clinic visit				
Government perspective	$2.21	$1.66	$2.76	[11, 34, 35]
Societal perspective	$3.20	$2.40	$4.00	
Cost per hospital admission				
Government perspective	$31.16	$23.37	$38.95	[11, 34, 45]
Societal perspective	$41.52	$31.14	$51.90	

In this analysis, we used data on access to healthcare from the 2014 Demographic and Health Survey, which reports a 45% proportion of children with diarrhea receiving care at a health facility [31]. We applied this proportion to non-severe and severe RVGE treated in outpatient and 80% to severe RVGE treated in inpatient, reflecting that not all severe cases may access inpatient care.

#### Vaccine efficacy, coverage, timeliness, and wastage

We assumed that ROTAVAC would maintain a health impact similar to ROTARIX. As a result, vaccine efficacy used in this analysis assumed similar protective efficacy and waning for all rotavirus vaccines evaluated. Efficacy values are based on Clark et al. data generated from a pooled analysis of all published rotavirus vaccines’ randomized control trials [32]. We used a 97% coverage rates for rotavirus vaccine based on the WHO and UNICEF estimates of national immunization coverage (WUENIC) reported for July 2019. For the first 12 months, coverage timeliness ranged from 0% to 67% for dose 1, 0% to 52% for dose 2, and 0% to 70% for dose 3 [31]. We did not account for COVID-19 impact on routine immunization in this analysis, this is because provision of essential health service continued in Ghana with ‘stay home order’ imposed on only two major cities Greater Accra and Greater Kumasi for three weeks. We consider this impact of COVID-19 on routine immunization to be negligible. In other words, coverage rate and timeliness values used should be considered optimistic.

We used a 5% vaccine wastage rate for ROTARIX, 10% for ROTAVAC 5-dose, and 15% for ROTAVAC 10-dose [17, 33]. Wastage rates values were informed by data from the Gavi detailed products profile document, from a wastage study undertaken recently in Ghana, and from information from the EPI program. To address the uncertainty around what wastage rate could be achieved with ROTAVAC and the potential implications on cost-effectiveness, we conducted a threshold analysis to determine at what wastage rate ROTAVAC 5-dose and ROTAVAC 10-dose achieved similar cost-effectiveness as ROTARIX. Table 1 shows the data inputs used for vaccine efficacy, coverage, timeliness, and wastage.

#### Health care costs for diarrhea treatment

Provider treatment cost data were taken from Ghana’s National Health Insurance Scheme (NHIS) 2019 tariff schedule for the treatment of diarrhea and vomiting and 2021 Medicines List tariff [34, 35]. Reimbursement rates for the same type of condition differ across providers based on the provider type/ownership and level—i.e., a public hospital receives a different rate from a private hospital for treating the same condition. Therefore, the average tariff across facility ownership and level of care (i.e., primary, secondary, tertiary) was used.

The treatment cost data are reported in 2019 Ghana cedis and the medicine list tariff is reported in 2021 Ghana cedis, however both tariffs were converted to 2020 US$ using mid-year exchange rates [36]. For household costs, we assume that the household share of total rotavirus costs is equivalent to the out-of-pocket cost share of Ghana’s total health expenditure. We obtained these national level metrics from the WHO Global Health Expenditure Database and then estimated the unknown household costs [11, 37]. Health care cost estimates are available in Table 1.

#### Vaccine program costs

Service delivery costs and switching costs were based on the primary data collection activities described earlier. For vaccine procurement costs, we used rotavirus vaccine prices published by UNICEF and calculated the country co-financing based on information from Gavi on Ghana co-financing level and transition trajectory for each of the three products assessed. The average price per course over the 10-year period was $5 for ROTARIX, $2.34 for ROTAVAC 5-dose, and $1.80 for ROTAVAC 10-dose. Ghana co-financing per course on average over 10 years was $3.54 for ROTARIX, $1.59 for ROTAVAC 5-dose, and $1.17 for ROTAVAC 10-dose [15, 41, 42]. We used this co-financing projection in our base case scenario, full vaccine prices for our high scenario, and developed a third scenario assuming Ghana’s transition from Gavi support would be at a slower pace than current projections (Table 1). Reported wastage data in addition to other parameters, including international handling and transportation, were used to model vaccine and commodities costs (Table 1).

#### Sensitivity analysis

To explore the impact of parameter uncertainty on the incremental cost-effectiveness ratio (ICER), we performed a one-way sensitivity analysis. Low and high values used in the sensitivity analysis and their references are available in Table 1.

We also performed a probabilistic sensitivity analysis (PSA) on a series of parameters, with 1,000 iterations to create a cost-effectiveness plane showing all iterations and median ICER for each product. Parameters included in the PSA were population data, disease burden, DALY weights, duration of illness, vaccine coverage, vaccine efficacy, vaccine waning, vaccine price, vaccine timeliness, incremental costs per dose, and healthcare costs.

#### Cost-effectiveness interpretation

We used a cost-effectiveness threshold of 0.5 times the Ghana GDP per capita following World Bank data which was $1,103 for the year 2020 to interpret our results [46]. Costs and health outcomes were discounted at 3% per year, and all costs are reported in 2020 US$.

### Ethical considerations

This study received ethical approval from the Ghana Health Service Ethics Review Committee (GHS-ERC 012/11/20) and Noguchi Memorial Institute for Medical Research Institutional Review Board (029/20-21).

## Results

### Incremental supply chain and service delivery cost for ROTARIX and ROTAVAC in 2020

The introduction of ROTAVAC did not result in any new purchases of cold chain equipment at any level of the supply chain. Only one district in the sample reported that a refrigerator had been provided to the facility because of ROTAVAC introduction. The system also had enough transport capacity to accommodate ROTAVAC. Therefore, there was no change in financial costs for vaccine transport; i.e., no change in delivery schedule, frequency of trips, or routes because of this switch because rotavirus vaccines were already in the EPI schedule and the schedule aligns with other vaccines already included in the schedule. Moreover, no healthcare workers were recruited as a result of the vaccine switch. In effect, no incremental financial costs were incurred for cold chain, transport, or human resources because of the switch to ROTAVAC.

We report 25^th^ and 75^th^ percentiles rather than minimum and maximum values as there were outliers caused by incomplete administrative data. Incremental economic costs for supply chain and service delivery were estimated to be $2.40 per dose for ROTARIX, $1.81 for ROTAVAC 5-dose, and $1.76 for ROTAVAC 10-dose as shown in Table 2. Although cost per dose is lower with ROTAVAC, when calculating for a full course, cost per course is lower for ROTARIX ($5.43 with ROTAVAC vs $4.80 with ROTARIX when excluding the vaccine price).

**Table 2:** Economic cost of supply chain and delivery cost per dose of rotavirus vaccines in 2020 US$.

Cost category	ROTARIX		ROTAVAC (5-dose)		ROTAVAC (10-dose)	
	Median ($)	25th and 75th percentile ($)	Median ($)	25th and 75th percentile ($)	Median ($)	25th and 75th percentile ($)
National	0.024	NA	0.017	NA	0.016	NA
Regional	0.01	0.007; 0.011	0.005	0.004; 0.006	0.005	0.004; 0.005
Directorate	0.14	0.08; 0.21	0.10	0.06; 0.14	0.09	0.06; 0.14
Health facility	2.23	1.10; 4.51	1.69	0.84; 3.04	1.65	0.82; 2.93
Incremental cost per dose	2.40	1.21; 4.74	1.81	0.92; 3.20	1.76	0.90; 3.09

### Cost of switching from ROTARIX to ROTAVAC in 2020

The financial costs associated with Ghana’s switch to ROTAVAC in 2020 amounted to $453,070, while economic costs amounted to $883,626. The switch costs were categorized according to the three administrative levels of the Ghanaian health system. Thus, for both financial and economic costs, the district level cost was the highest, contributing $217,140 (48%) and $583,932 (66%), respectively, to the total costs. The national level contributed 28% to financial cost and 15% to economic costs, while regional level contributed 24% to financial cost and 19% to economic costs (Table 3). Overall, the major cost driver for the switch was training, contributing to $351,649 (77.6%) of total financial cost and $576,353 (65.2%) of total economic costs. Social mobilization and IEC was the second largest financial cost driver (15.7%), but for economic cost it was the smallest cost driver (9.5%). Stakeholder engagement workshops recorded a financial cost of $20,124 (4.4%) and total economic cost of $130,298 (14.7%) (Table 2).

**Table 3:** Total financial and economic cost of the switch from ROTARIX to ROTAVAC in 2020 US$.

Cost item	Financial cost ($)					Economic cost ($)				
	National	Regional	District	Sub-total	% of total	National	Regional	District	Sub-total	% of total
Planning and coordination committees	6,800	3,163	0	9,963	2.2	8,078	10,745	73,797	92,620	10.5
Stakeholders’ engagement workshops	0	0	20,124	20,124	4.4	460	769	129,069	130,298	14.7
Training	120,996	70,073	160,580	351,649	77.6	125,000	106,723	344,630	576,353	65.2
Social mobilization and IEC	0	34,898	36,436	71,334	15.7	0	47,919	36,436	84,355	9.5
**Total**	**127,796**	**108,134**	**217,140**	**453,070**	**100**	**133,538**	**166,156**	**583,932**	**883,626**	**100**

### Cost-effectiveness of ROTARIX and ROTAVAC over the period 2020 to 2029

The results show that rotavirus vaccination has significant health impact, reducing rotavirus-related mortality and illness among children younger than five years old. Estimates of health impact in this analysis were of similar magnitude to the original CEA. Rotavirus vaccination averted more than 1.8 million RVGE cases, 116,521 hospitalizations, and 6,471 deaths, over a ten-year period. A total of 167,000 DALYs (discounted) were averted. Table 4 shows that for ROTARIX, ROTAVAC 5-dose, and ROTAVAC 10-dose, continued vaccination is expected to avert approximately $4.7million and $6.4 million healthcare cost from the government and societal perspectives, respectively. The costs averted due to vaccination are higher from the societal perspective because of the inclusion of averted household out-of-pocket costs. The total cost of the vaccination program over the 2020 to 2029 period is approximately $66.5 million with ROTARIX, $56.2 million with ROTAVAC 5-dose, and $52 million with ROTAVAC 10-dose (Table 4).

**Table 4:** Health impact, costs, and cost-effectiveness of vaccination over 10 years starting from 2020.

Parameter	Deterministic result
Number of immunized children	8,196,301
RVGE cases averted	1,765,564
RVGE visits averted	794,504
RVGE hospitalizations averted	116,521
RVGE deaths averted	6,471
DALYs averted (discounted)	166,935
Government treatment costs averted	$4,691,727
Societal treatment costs averted	$6,428,238
Vaccine program cost (discounted)	
With ROTARIX	$66,528,402
With ROTAVAC 5-dose	$56,227,006
With ROTAVAC 10-dose	$51,995,896
Cost per fully immunized child	
With ROTARIX	$8.10
With ROTAVAC 5-dose	$6.90
With ROTAVAC 10-dose	$6.30
Cost per DALY averted (government perspective) compared to no vaccination	
ROTARIX	$370
ROTAVAC 5-dose	$309
ROTAVAC 10-dose	$283
Cost per DALY averted (societal perspective) compared to no vaccination	
ROTARIX	$360
ROTAVAC 5-dose	$298
ROTAVAC 10-dose	$273
Cost per DALY averted (government perspective) compared to ROTARIX	
ROTAVAC 5-dose	Cost-saving
ROTAVAC 10-dose	Cost-saving
Cost per DALY averted (societal perspective) compared to ROTARIX	
ROTAVAC 5-dose	Cost-saving
ROTAVAC 10-dose	Cost-saving

Table 4 further shows that the incremental cost per fully immunized child is between $6.30 and $8.10 with ROTAVAC 10-dose being the lowest and ROTARIX being the highest cost. Again, from the government perspective the costs per DALY averted are $370 for ROTARIX, $309 for ROTAVAC 5-dose, and $283 for ROTAVAC 10-dose. From both the government and societal perspectives, cost per DALY averted for ROTAVAC (5-dose and 10-dose) are more favorable and cost-saving compared to ROTARIX.

### Sensitivity analysis

The one-way sensitivity analysis shows that parameters related to disease burden and vaccine efficacy were the most influential on results (see S1 Fig).

**Fig 1:**
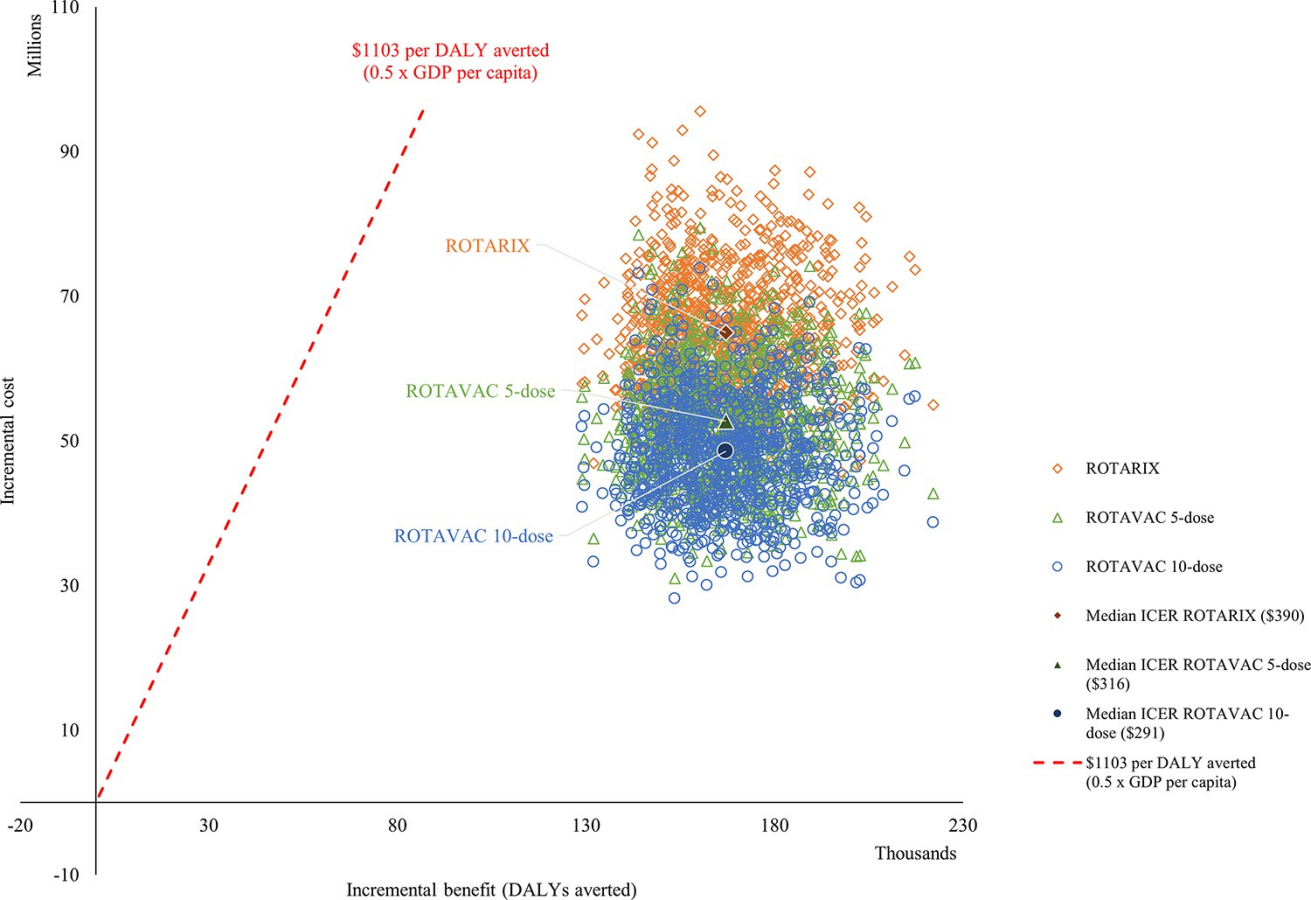
PSA results for ICER from the government perspective. Each dot on Fig 1 represents the ICER resulting from one of the thousands run completed for the probabilistic sensitivity analysis. The three highlighted plain dots represent the mean ICER for each product evaluated. The red dotted line represents the cost-effectiveness thresholds used to interpret results.

The PSA results show that at any point in time, the median ICERs are below the threshold. Also, in all iterations, ROTAVAC 10-dose has the most desirable ICER ($291). Fig 1 displays the results in terms of discounted cost per DALY averted for PSA.

**Fig 2:**
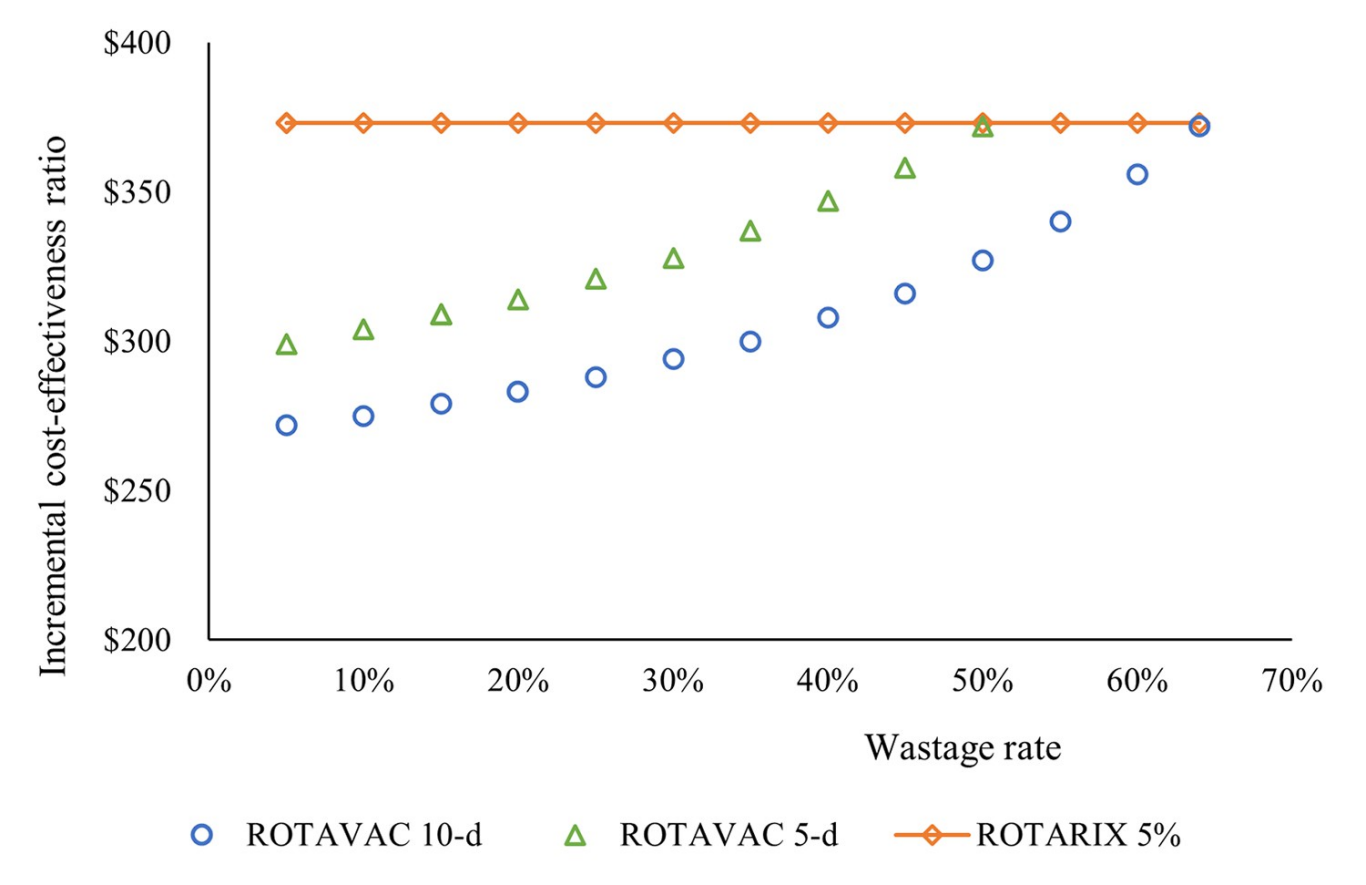
Wastage rate threshold analysis. ROTAVAC 10-dose would need to have wastage exceeding 60% for ROTARIX to be the most favored product.

Fig 2 shows the wastage rate threshold analysis. To achieve cost-effectiveness similar to ROTARIX, we found that the wastage rate has to reach 50% for ROTAVAC 5-dose and 64% for ROTAVAC 10-dose before the ICERs can be equal to the ROTARIX ICER.

## Discussion

We evaluated the economic implications of the switch from ROTARIX to ROTAVAC in Ghana. The results show that, assuming the health impact remains similar, ROTAVAC presents an economic advantage over ROTARIX and switching products was a cost-saving decision from both the government and societal perspectives. A similar policy shift in Palestine from ROTARIX to ROTAVAC was previously reported to be cost-saving [20]. The main driver for these results is a lower price per dose for ROTAVAC compared to ROTARIX which outweighs any cost disadvantages resulting from having to deliver an additional dose of vaccine. Even more revealing is the ROTAVAC 10-dose presentation, which presents additional economic advantages over the 5-dose presentation.

The switch required resources for planning and coordination, training, stakeholder engagement, social mobilization, and IEC. Those resources, which amounted to less than $500,000 for financial costs and around $900,000 for economic costs, represented a lower cost than the original introduction costs in terms of supply chain and service delivery [47]. The cost to deliver a dose of ROTAVAC is lower than for a dose of ROTARIX, however, the cost to deliver a full course of ROTAVAC is higher, because of the third dose required, which is not needed as part of the ROTARIX schedule. The supply chain and service delivery cost per dose was larger at the lower levels of the supply chain because of the relatively higher fixed costs for cold chain and health workers’ time spent on service delivery, relative to the volume of vaccines used.

The total net undiscounted financial cost of rotavirus vaccination with ROTAVAC 5-dose, currently in use in Ghana, is estimated to represent $1.61 million on average per year. This represents 0.70% of Ministry of Health’s budget for goods and services in 2020 and 2.92% of total immunization costs for 2019, which makes rotavirus vaccination likely affordable [48, 49].

One of the main concerns regarding the switch to ROTAVAC was the level of vaccine wastage that would be achieved with a multidose vial presentation. This aspect is even more critical when considering ROTAVAC 10-dose. While the wastage rate of both ROTAVAC 5-dose and 10-dose are higher than ROTARIX, their ICERs remain more favorable. The threshold analysis on wastage rate indicated that the ROTAVAC ICER would remain favorable compared to ROTARIX even at very high wastage rates. These high wastage rates are unlikely to be realized as similar multi-dose products typically have wastage rates lower than 30% [17, 50, 51]. Further, the labeling change for ROTAVAC which allows remaining doses in opened vials to be stored and used within a 28-day period, which was approved by WHO in 2021, will further help to reduce vaccine wastage and increase the economic advantage provided by this product [51].

This study updated an existing CEA that was conducted for rotavirus vaccination in Ghana using ROTARIX. The findings show relatively similar health impact of vaccination with small differences observed between this current study and the original CEA. The small changes that are observed are attributed to the use of updated population projection data, updated RVGE mortality rates, and updated disease age distribution.

Over the next 10 years, the vaccination program is expected to be cost-effective at 0.5 times Ghana’s GDP per capita ($1,103) even during the period when support from Gavi declines and Ghana transitions to fully self-financing vaccines. Even though, the current threshold shows that the vaccination program in the next 10 years will be cost-effective, others have recommended a more stringent threshold for Ghana, which is 0.39 times GDP per capita, yet the program remains cost-effective for this threshold [52]. Results from the societal perspective show even higher savings on healthcare costs from household-borne costs, which is evidence of the economic burden presented by rotavirus for families and the health system of Ghana. The results of this analysis are consistent with previous studies in other LMICs, which found rotavirus vaccination programs compared to no vaccination to be highly cost-effective [8, 18, 26, 53, 54]. Two previous studies in Ghana have reported rotavirus vaccination to be highly cost-effective [11, 55]. However, the current study addresses the gap in those previous studies: one studied only ROTARIX and the other made assumptions around cost to deliver additional products available. In this study, we evaluated different products, using primary data to inform cost to deliver each vaccine.

Economic analyses projecting the costs and benefits of programs can be effective tools to aid decision-making. Notwithstanding, we acknowledge some limitations in this analysis. UNIVAC, as a static model, does not account for indirect effects of vaccination i.e., herd effects. However, there is substantial uncertainty about the potential scale of these effects in LMICs [56]. Had we incorporated these effects, our cost-effectiveness estimates would have been more favorable to rotavirus vaccination, but our conclusions about the relative cost-effectiveness of each product would be unchanged. Substantial efforts were made to ensure the model was populated with the best available data, and the results have been further examined through a sensitivity analysis. A few areas with limited data should be noted. First, our data for cost of care reflects recent NHIS tariffs in Ghana. Tariffs may not be a perfect representation of the costs of care but it has been used in a similar study in Ghana [11] which admitted it to be a fair representation of other estimates in Northern Ghana and the international literature. For supply chain and service delivery cost, though our sampling is covering districts and regions of the three ecological zone, it was not designed to be entirely representative as we were limited by budget in the number of sites for data collection. For switch costs, we extrapolated regional and district costs. We used simple PERT-Beta distributions for our PSA using readily available information on the central estimate and range for each input parameter. More realistic distributions could theoretically have been assigned to each input. This would not have altered our central results but is a limitation in our estimates of probabilistic uncertainty [57].

One of the main assumptions in this analysis was the use of the same efficacy level for all three vaccines, which resulted in identical health impact. The availability of efficacy data for ROTAVAC is limited, with only one study in India which found efficacy levels to be 56% in children 12 months old [58]. However, it is difficult to directly translate this efficacy level to the Ghanaian context.

## Conclusion

Findings from this study provide key stakeholders in Ghana with evidence that supports their decision to switch from ROTARIX to ROTAVAC in the rotavirus vaccination program. Moreover, the analysis indicates that, in the next decade, should the government of Ghana switch to the ROTAVAC 10-dose presentation, there will be further cost savings to the health system and further economic benefits if the country implements the open vial policy. The analysis also provides economic evidence to other countries currently exploring the possibility of switching to a different rotavirus vaccine product. Our analysis suggests that vaccine price is a critical parameter and, therefore, the availability of low-cost products is an incentive to reevaluate vaccine choices. Overall, rotavirus vaccination is cost-effective at 0.5 times Ghana’s GDP per capita and has significant health and economic impact in the Ghanaian health system.

## Supporting information

S1 Fig Results of one-way sensitivity analysis. (PDF)
