# Supplementary figures and images for: Rotavirus vaccine product switch in Ghana: An assessment of service delivery costs, switching costs, and cost-effectiveness
Source: PLOS Glob Public Health. 2023 Aug 9;3(8):e0001328. doi: 10.1371/journal.pgph.0001328 (PMC10411789; doi:10.1371/journal.pgph.0001328)

**Supplementary Figure 1. Results of one-way sensitivity analysis.**

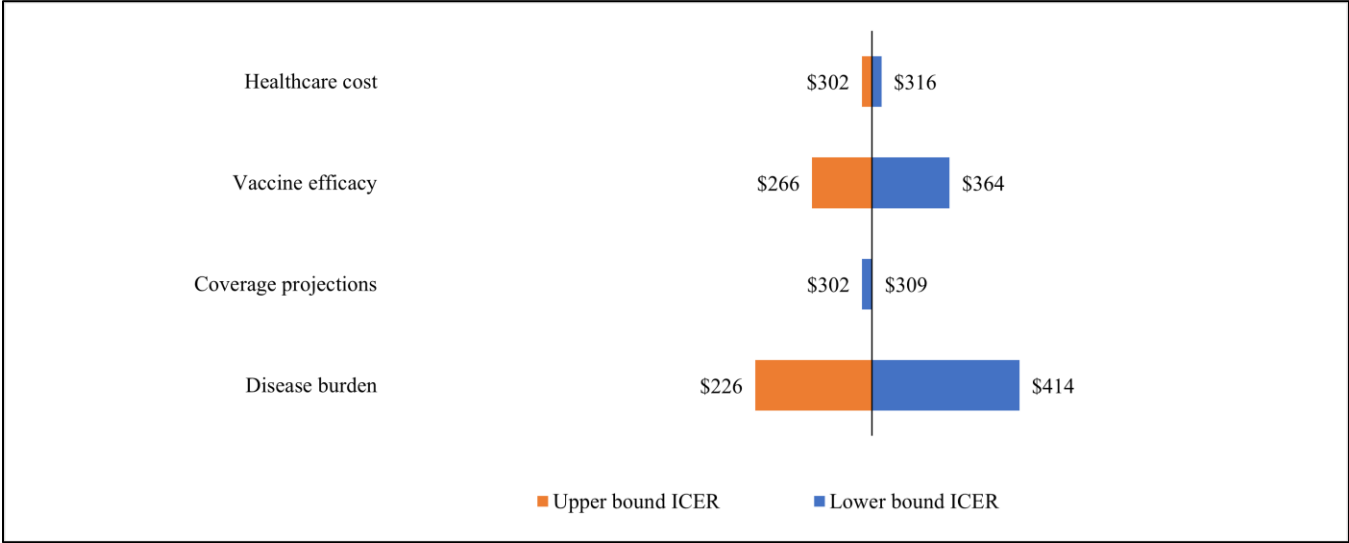

Supplement: S1 Fig — (PDF) [file pgph.0001328.s001.pdf]
